# Deep learning-based detection of functionally significant stenosis in coronary CT angiography

**DOI:** 10.3389/fcvm.2022.964355

**Published:** 2022-11-15

**Authors:** Nils Hampe, Sanne G. M. van Velzen, R. Nils Planken, José P. S. Henriques, Carlos Collet, Jean-Paul Aben, Michiel Voskuil, Tim Leiner, Ivana Išgum

**Affiliations:** ^1^Department of Biomedical Engineering and Physics, Amsterdam University Medical Center, University of Amsterdam, Amsterdam, Netherlands; ^2^Amsterdam Cardiovascular Sciences, Heart Failure and Arrhythmias, Amsterdam, Netherlands; ^3^Informatics Institute, University of Amsterdam, Amsterdam, Netherlands; ^4^Department of Radiology and Nuclear Medicine, Amsterdam University Medical Center, University of Amsterdam, Amsterdam, Netherlands; ^5^AMC Heart Center, Amsterdam University Medical Center, University of Amsterdam, Amsterdam, Netherlands; ^6^Onze Lieve Vrouwziekenhuis, Cardiovascular Center Aalst, Aalst, Belgium; ^7^Pie Medical Imaging BV, Maastricht, Netherlands; ^8^Department of Cardiology, University Medical Centre Utrecht, Utrecht, Netherlands; ^9^Department of Radiology, University Medical Center Utrecht, Utrecht, Netherlands; ^10^Department of Radiology, Mayo Clinic, Rochester, MN, United States

**Keywords:** convolutional neural networks, coronary computed tomography angiography, fractional flow reserve, transformer, coronary artery tree

## Abstract

Patients with intermediate anatomical degree of coronary artery stenosis require determination of its functional significance. Currently, the reference standard for determining the functional significance of a stenosis is invasive measurement of the fractional flow reserve (FFR), which is associated with high cost and patient burden. To address these drawbacks, FFR can be predicted non-invasively from a coronary CT angiography (CCTA) scan. Hence, we propose a deep learning method for predicting the invasively measured FFR of an artery using a CCTA scan. The study includes CCTA scans of 569 patients from three hospitals. As reference for the functional significance of stenosis, FFR was measured in 514 arteries in 369 patients, and in the remaining 200 patients, obstructive coronary artery disease was ruled out by Coronary Artery Disease-Reporting and Data System (CAD-RADS) category 0 or 1. For prediction, the coronary tree is first extracted and used to reconstruct an MPR for the artery at hand. Thereafter, the coronary artery is characterized by its lumen, its attenuation and the area of the coronary artery calcium in each artery cross-section extracted from the MPR using a CNN. Additionally, characteristics indicating the presence of bifurcations and information indicating whether the artery is a main branch or a side-branch of a main artery are derived from the coronary artery tree. All characteristics are fed to a second network that predicts the FFR value and classifies the presence of functionally significant stenosis. The final result is obtained by merging the two predictions. Performance of our method is evaluated on held out test sets from multiple centers and vendors. The method achieves an area under the receiver operating characteristics curve (AUC) of 0.78, outperforming other works that do not require manual correction of the segmentation of the artery. This demonstrates that our method may reduce the number of patients that unnecessarily undergo invasive measurements.

## 1. Introduction

Coronary artery disease (CAD) is the leading cause of death worldwide (1, 2). CAD is characterized by a buildup of atherosclerotic plaque in the coronary arteries, potentially leading to a functionally significant stenosis, i.e., stenosis that causes myocardial ischaemia. Currently, invasive fractional flow reserve (FFR) measurements are considered the clinical reference for determining the functional significance of a stenosis. However, invasive FFR is associated with high costs and it constitutes a burden for the patient (3, 4). Hence, identifying patients with functionally significant stenosis prior to the invasive measurements and treatment would be of high value. While visual interpretation of coronary CT angiography (CCTA) by clinical experts enables identification of the vast majority of functionally significant stenoses (high sensitivity), it suffers from a high number of false positives (low specificity) (5, 6). As a consequence, 20–50% of invasive FFR measurements are performed unnecessarily (6). Therefore, predicting FFR non-invasively from CT angiography is a subject of intensive investigations.

For non-invasive FFR prediction from CCTA, several algorithms have been proposed. Currently, most accurate methods are based on computational fluid dynamics (CFD) (7–12). However, CFD-methods are computationally expensive, hampering (real-time) implementation on clinical workstations. Moreover, CFD-based methods rely on the accuracy of the anatomical artery tree model, i.e., artery lumen segmentation and boundary conditions describing aortic pressure and peripheral resistances, which are challenging to obtain.

In addition to development of CFD-based FFR prediction methods, approaches emerged that correlate quantitative indices derived from CCTA with measured FFR value. These clinical indices characterize a coronary artery through e.g., transluminal attenuation gradient (TAG) (13, 14) or plaque volume (15, 16), or describe specific lesions by quantifying degree of stenosis (16, 17) or contrast density difference (CDD) (18, 19). While the mathematical simplicity and intuitive design of the calculated indices enables their interpretation, it limits their capability to model the complex relationship between FFR and the coronary artery characteristics on CCTA. Hence, to improve FFR prediction with clinical indices, machine learning classifiers were employed that combined multiple indices (11, 16, 20–24). This led to a substantial performance increase compared to the performance of a single index. Similarly, using clinical indices describing the local geometry and plaque composition, as well as global features describing the entire artery tree, Itu et al. (21) trained a deep learning classifier for prediction of the pressure gradient caused by each lesion. For training, the authors leveraged hemodynamic simulations in 12,000 artificial coronary anatomies. To enable learning of relationships between lesions, Wang et al. (25) and Gao et al. (26) employed the same features as input to a recursive neural network (RNN). However, these index-based works share a drawback with CFD-based methods: calculating the indices requires accurate segmentation of the coronary artery lumen, which can be highly challenging, especially in the presence of pathology (27). While these methods typically use an automatic segmentation method as a starting point, errors in the automatic segmentation regularly necessitate substantial manual interaction.

To avoid lengthy assessment times, algorithms that apply deep learning technology directly to the CCTA scan have been investigated. Deep learning algorithms have shown the ability to model complex relations of image characteristics in a large number of medical task (28). However, these methods often require a large amount of diverse training data, which may be challenging to obtain in the medical domain. Hence, previous deep learning-based works reduced the complexity of the task by focusing analysis to a relevant region of interest (29–32) or by training separate networks to extract image characteristics (29, 31–33). Given that obstruction in the coronary arteries is expected to lead to underperfusion of the left ventricle (LV), Zreik et al. (29) focused analysis on the LV myocardium by characterizing it using a convolutional autoencoder (CAE). Subsequently, the authors predicted the presence of a functionally significant stenosis using a support vector machine (SVM), which can be strained with limited data due to its small number of parameters. In a subsequent study, Zreik et al. (31) characterized the coronary arteries by training a CAE on multi planar reconstructions (MPRs) of the coronary arteries. Related to this, Denzinger et al. (30) used a CNN in combination with an RNN to classify MPRs. The authors used the clinical revascularization decision as reference label, obtained using functional tests including cardiac stress MRI or MIBI SPECT. To further improve performance, Zreik et al. (32) combined the characterizations of the myocardium and the coronary arteries using a deep learning-based multi instance learning framework. As an alternative to focusing analysis to a region of interest, Kumamaru et al. (33) enhanced lumen-related image features using a difference image between the CCTA scan and a non-contrast cardiac CT, synthesized from CCTA using deep learning. Thereafter, authors trained a 3D ladder network to extract relevant image characteristics. These deep learning-based works were limited by their moderate performance. Unlike other deep learning-based works that applied CNNs to the CCTA scan, Li et al. (34) first used the artery segmentation to extract a point cloud representing the coronary artery geometry. The authors used this point cloud as input to a modified version of the point-net (35), to predict the pressure in the coronary artery tree. However, the authors used hemodynamic simulations as reference labels in training and testing and hence, the performance compared to invasive FFR measurements is unknown.

In this work, we propose a method to non-invasively predict the presence of a functionally significant stenosis in an artery through deep learning-based analysis of CCTA scans. As in previous deep learning works, we focus on a region of interest by first extracting an MPR for the artery of interest. Given that previous research demonstrated the importance of lumen area, its attenuation and plaque volume for predicting FFR, we exploit these characteristics. To circumvent the need for challenging lumen segmentation, during testing, we use a convolutional neural network (CNN) to directly extract these characteristics from the MPR along the artery centerline. Additionally, we extract characteristics directly from the coronary artery tree that indicate per coronary artery centerline point whether it is located in a main artery or side-branch and whether a bifurcation is present at that location. Thereafter, using the extracted characteristics we assess the functional significance of FFR.

For this purpose, we train a second network to perform both regression of the FFR value and classification of the functional significance of an artery. In contrast to previous works that use abstract, high dimensional features, extraction of our specific characteristics is supervised, resulting in targeted information distillation and lower dimensional features. While training of previous deep learning-based works on the limited training data requires compressing the high dimensional features along the artery prior to training the stenosis classification (31, 32), our targeted extraction of artery characteristics enables us to directly use these characteristics along the artery as input to our second network. This second network is designed to exploit the spatial structure encoded in the extracted characteristics through the use of convolutions and self-attention. The so-learned representations are likely more descriptive than unsupervised features characterizing the entire artery. Additionally, using tangible characteristics, instead of the abstract features employed in previous deep learning works (29, 31–33), enables interpretability of our method. We performed experiments on a diverse data set from multiple centers and vendors.

This paper is organized as follows. The data is described in Section 2. Section 3 provides a description of the method, which is followed by a description of our evaluation in Section 4 and by experiments and results in Section 5. We discuss our findings in Section 6 and describe our conclusions in Section 7.

## 2. Data

### 2.1. Patients and imaging data

This study retrospectively included 657 patients who underwent CCTA for suspected obstructive CAD. Scans were acquired in three different hospitals: Scans of 263 patients (age 47–79 years) were acquired in the Onze Lieve Vrouwe Ziekenhuis, Aalst, Belgium (Site 1) with a Siemens Somatom Definition Flash CT scanner; Scans of 152 patients (age 34–84 years) were acquired in the University Medical Center Utrecht, the Netherlands (Site 2) with a Philips iCT 256 CT scanner; Scans of 243 patients (age 48–85 years) were acquired in the Amsterdam University Medical Centers—location University of Amsterdam, the Netherlands (Site 3) with a Siemens Somatom Force CT scanner. Patients were only included if all arteries were in the field of view of the CCTA scan. This study was approved (Site 1) or the need for informed consent was waived by the respective institutional review boards (Site 2, Site 3).

During acquisition, contrast medium was injected with a flow rate of 4 to 6 mL/s for a total of 30 to 92 mL iopromide (Ultravist 300 mg I/mL, Bayer Healthcare, Berlin, Germany), depending on the patient weight and test bolus images (29, 36). The tube voltages ranged between 70 and 140 kVp and tube currents between 71 and 901 mAs. All scans were reconstructed to an in-plane resolution ranging from 0.22 to 0.83 mm^2^ with 0.3 to 0.5 mm slice increment and 0.5 to 1.0 mm slice thickness.

In total 85 out of 658 patients were excluded because the quality of the CCTA scan was not sufficient due to e.g., severe step-and-shoot artifacts (*n* = 22), severe cardiac motion artifacts (*n* = 47) or artifacts caused by metal implants (*n* = 16; Table 1). Furthermore, patients who underwent stenting or coronary artery bypass grafting (CABG) prior to CCTA acquisition were excluded (*n* = 4). After exclusions, 569 patients remained for further analysis.

**Table 1:** Patients were excluded due to artifacts, i.e., severe step-and-shoot artifacts, severe cardiac motion artifacts or artifacts caused by metal implants.

	**Artifacts**			**Stenting/**	**Total**
	**Step and shoot**	**Motion**	**Metal**	**CABG**	**excluded**
Site 1	13	21	2	1	37
Site 2	8	15	11	3	37
Site 3	1	11	3	0	15
Total	22	47	16	4	89

For development and validation of the method, 438 arteries with FFR measurements from 302 patients were used. Additionally, for independent evaluation of the method, the performance was evaluated with two held-out test sets. The first set consisted of 76 arteries with FFR measurements in 67 patients randomly sampled from all three sites. It is referred to as Test_Cath_. The sets used for development and validation, as well as Test_Cath_, consist of patients with intermediate degree of anatomical stenosis for which the cardiologist recommended invasive FFR measurement to assess hemodynamic significance of the stenosis. Therefore, these sets are representative of the clinical population that undergoes FFR measurement for suspicion of obstructive CAD in the catheterization laboratory, which represents our primary target population. The second test set consisted of 600 arteries of 200 patients, in which instead of invasive FFR measurement obstructive CAD was ruled out as they were assigned to category zero (absence of stenosis or plaque in all coronary arteries) or one (low degree of anatomical stenosis or plaque in all coronary arteries) according to the Coronary Artery Disease-Reporting and Data System (CAD-RADS) (37). Hence, arteries in this population have a degree of stenosis < 25%. The chances of finding functionally significant stenosis in these patients would be marginal (38). To warrant that our algorithm classifies these arteries correctly, they were used for testing by assuming FFR >0.8, indicating the absence of functionally significant stenosis. Thus, this second test set is referred to as Test_NoCath_ and it is used for evaluation purposes only as no patient with little or no stenosis was sent for invasive FFR measurement.

**Figure 1:**
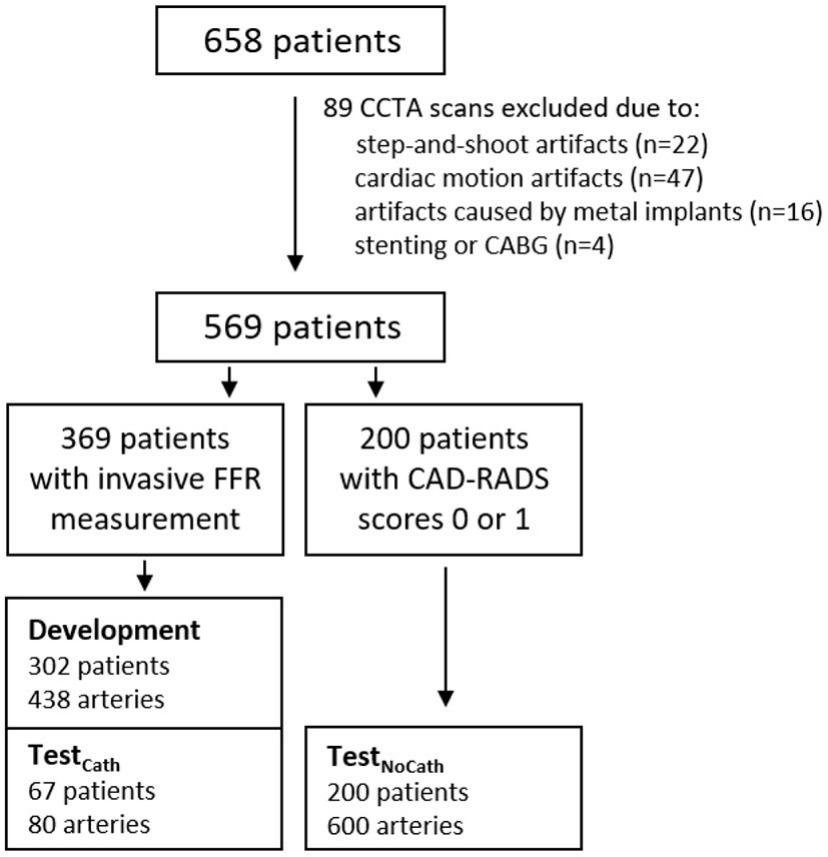
Data included in the study.

Analysis in this set was performed for the main arteries, i.e., left anterior descending artery (LAD), left circumflex artery (LCX) and right coronary artery (RCA). CAD-RADS scoring was performed within 3 days of the acquisition of the CCTA scan. Figure 1 and Table 2 show details regarding the data selection.

**Table 2:** Data set for training and validation (Development), and two separate test sets, one with FFR measurements (Test_Cath_) and one without (Test_NoCath_).

		**Development**	**Test_Cath_**	**Test_NoCath_**
Patients		302	67	200
**Arteries**		438	76	600
Hospital				
	Site 1	249	58	0
	Site 2	159	14	0
	Site 3	30	4	600
Anatomical segment				
	LAD	221	50	200
	LCX	81	9	200
	RCA	72	10	200
	LM	14	1	0
	SB	50	6	0
FFR statistics				
	% positive	0.42	0.78	0.00
	Mean FFR	0.82	0.70	-
	Std FFR	0.11	0.16	-

In addition to the number of arteries and patients in each set, the table lists the contribution of each site to each set, the anatomical segments in which the invasive measurements were performed and statistics describing the distribution of FFR values. Anatomical segments were categorized into the main branches, i.e., LAD, left anterior descending artery; LCX, left circumflex artery; RCA, right coronary artery; LM, left main; SB, side-branch of the main arteries.

### 2.2. FFR measurements

Among the 569 patients, 369 underwent invasive FFR measurement in 514 arteries. To measure FFR, a coronary pressure guidewire (Certus Pressure Wire, St. Jude Medical, St. Paul, Minnesota or Pressure wire X, Abbott Vascular, California) was inserted into the distal segment of the coronary vessel, and maximal hyperemia was induced by administration of intravenous adenosine through a central vein. The lowest FFR value measured at the most distal location was chosen for analysis. An FFR pullback was performed to assess the presence of drift. If multiple FFR measurements were available in one artery, the value measured at the most distal location was chosen. The maximum time interval between the acquisition of the CCTA scan and the FFR measurement was 90 days for Site 1 and 1 year for Site 2 and Site 3.

### 2.3. Reference artery characteristics

To train the network for extraction of artery characteristics, reference annotations of the coronary artery lumen and coronary calcium were required. Given the extensive manual workload of the tasks, these were performed semi-automatically in a subset of 56 arteries, randomly selected from the development data set. First, automatic segmentations of the lumen and calcium were generated in the original CT image volumes using methods previously developed in our group (39, 40). Thereafter, automatic segmentations were transferred to the MPR of the artery, visually inspected and corrected when needed. Using the segmentations, the reference lumen area and calcium area were generated by summing up the pixels of the respective segmentation in each cross-sectional slice of the MPR perpendicular to the artery centerline. Note that MPRs for all arteries share the same spacings and in-plane resolution. For the average lumen attenuation, the average of the image pixels within the lumen segmentation mask was calculated in each cross-sectional slice of the MPR.

## 3. Methods

**Figure 2:**
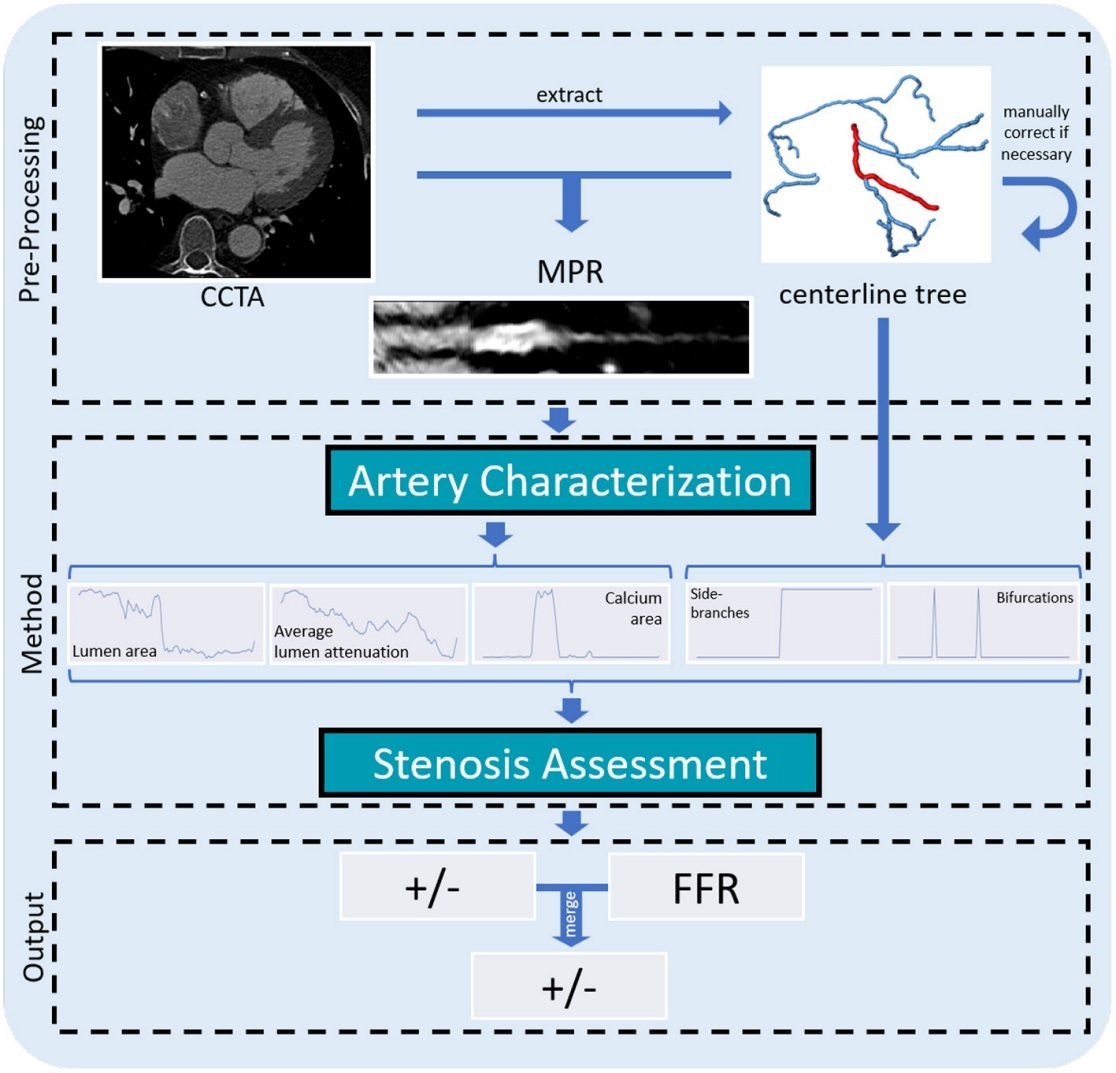
Overview of our method for assessing the presence of a functionally significant stenosis in a coronary artery. From the CCTA scan, we extract a coronary artery centerline tree. For each artery we generate an MPR that is further analyzed to predict the lumen area, its average attenuation and the calcium area per centerline point. These characteristics, as well as characteristics indicating the presence of bifurcations and whether the artery is a main branch or to a side branch of a main artery, are fed to the classification network to determine the presence of a functionally significant stenosis in the artery.

Our method assesses the functional significance of stenosis in an artery from CCTA. First, we extract the coronary artery centerline tree. To analyze the artery of interest, we then reconstruct an MPR. Subsequently, we extract relevant characteristics of the artery along its centerline using a 2D CNN and the characteristics of the artery within the coronary artery tree. Using these characteristics, we assess the presence of a functionally significant stenosis with a dedicated CNN (Figure 2).

### 3.1. Artery extraction

**Figure 3:**
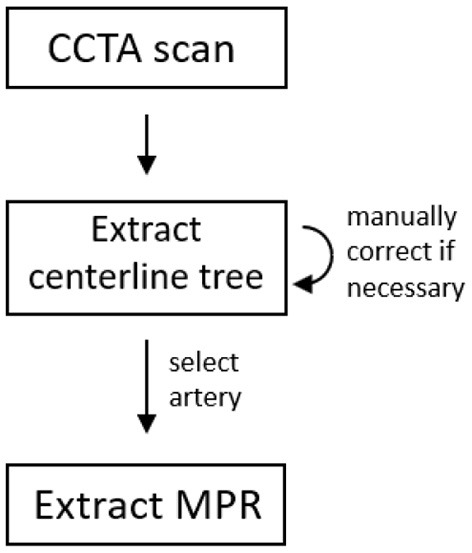
Pre-processing steps.

To localize the coronary arteries in the CCTA image, the coronary artery centerline tree is extracted and anatomical labels are assigned to the tree's segments using our previously developed method (41). Thereafter, the labeled centerline tree is inspected and manually corrected if needed. This is the only manual interaction that might be required for our method at test time. Figure 3 illustrates the pre-processing steps. In most cases this took 1 min, but could take up to at most 5 min when challenged by pathology. For each selected artery centerline, an MPR with 0.1 mm in-plane voxel size and 0.5 mm distance between MPR slices is reconstructed using trilinear interpolation. The in-plane shape of the MPR is 127 x 127 and the number of slices is dependent on the artery length. Finally, image intensities in the MPRs are normalized to zero mean and unit variance across the data set, to ensure training stability of the neural networks.

### 3.2. Artery characterization

#### 3.2.1. Extraction of coronary artery characteristics

**Figure 4:**
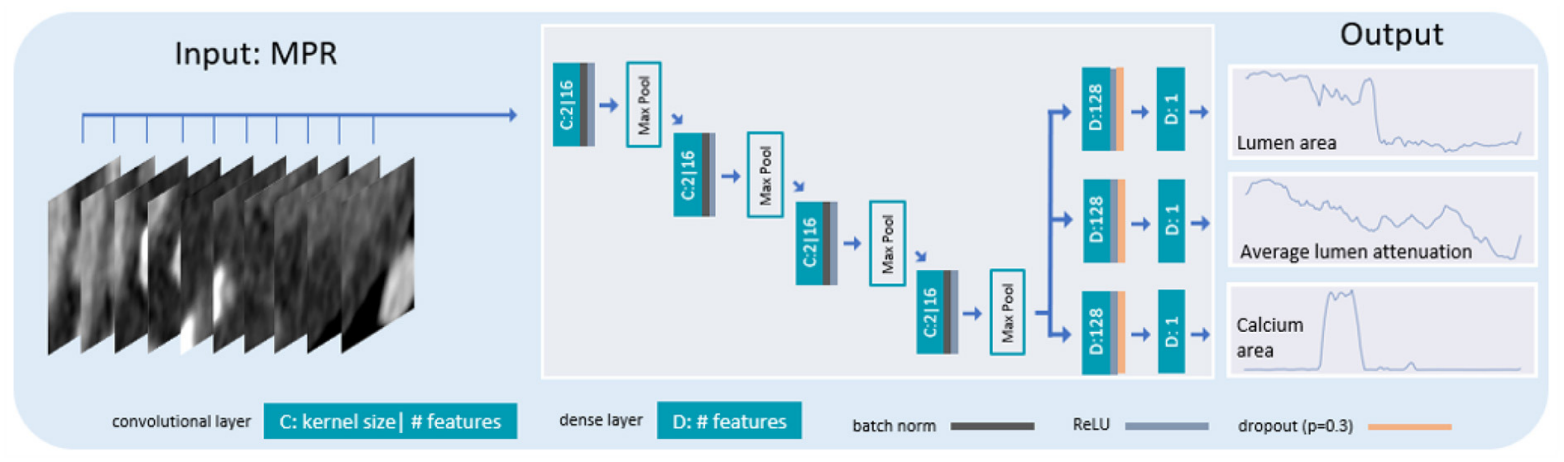
Architecture of the network for extracting artery characteristics. Stacks of 3 cross-sectional artery slices are fed to a 2D CNN with 4 pooling layers interleaved with convolutions. The network is trained to predict the lumen area, its average attenuation and the calcium area for the central slice of the 3 input slices.

To automatically characterize a coronary artery, we extract the lumen area, its attenuation and the amount of coronary artery calcium from the artery's MPR. Specifically, for each point of the coronary artery centerline, we predict the lumen area, the average lumen attenuation and the calcium area in its cross-section with a 2D CNN (Figure 4). The network analyzes stacks of three cross-sectional slices and consists of four alternating convolutional blocks and pooling operations. The convolutional blocks are comprised of two convolutional layers (kernel size 3, 16 filters), each followed by batch normalization and the ReLU activation function. Finally, three separate output heads regress values for the lumen area, average attenuation in the lumen and calcium area for the central slice of the input stack.

#### 3.2.2. Extraction of coronary tree characteristics

The coronary artery geometry has impact on the characteristics of the blood flow and local appearance of the artery. Therefore, for each point along the coronary artery centerline, we extract two additional characteristics. The first one indicates the presence of bifurcations at the artery centerline point. The second one indicates whether a centerline point belongs to a main branch (i.e., left main (LM), LAD, LCX, RCA) or a side-branch. The locations of bifurcations and side-branches follow from the tree topology and labels. Specifically, for each MPR slice, information about bifurcations and side branches was extracted from the coronary artery centerline point of the tree at that location, i.e., by considering the amount of successive centerline points and the label, respectively. We normalize all characteristics to zero mean and unit variance across the training data set.

### 3.3. Stenosis assessment

**Figure 5:**
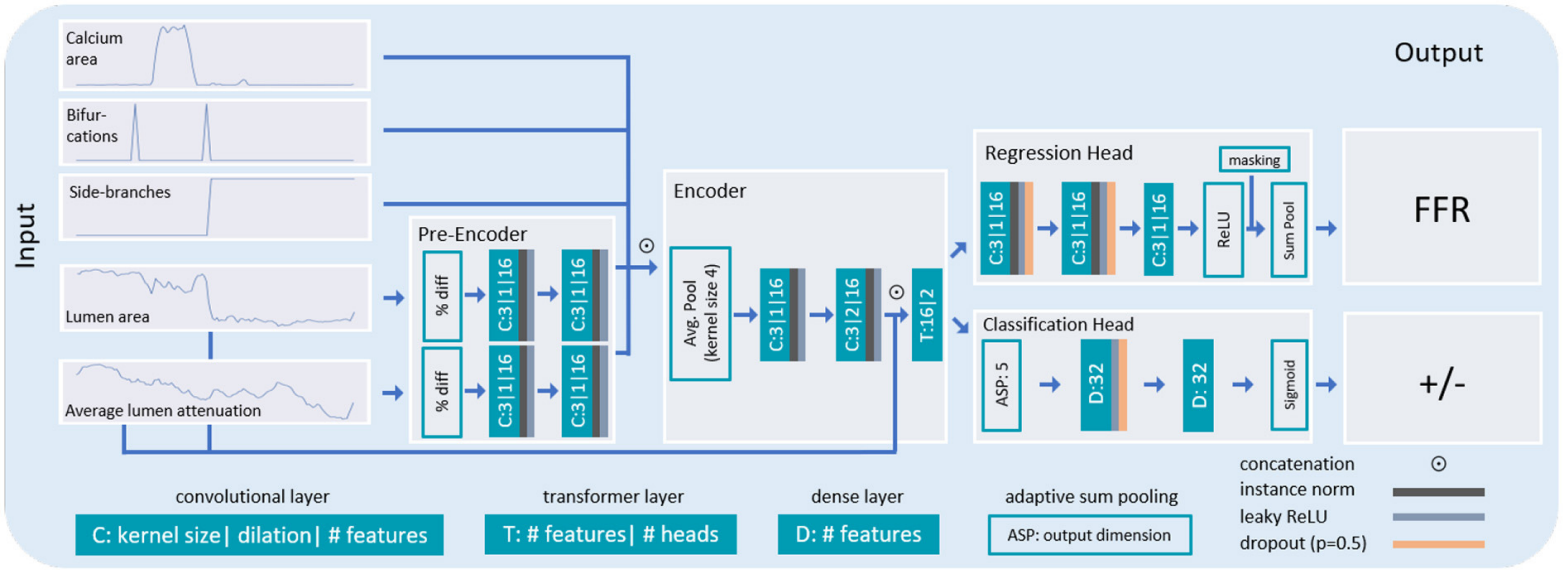
Architecture of the network used for stenosis assessment. The lumen area and its attenuation predicted by the characterization network are first pre-encoded and subsequently concatenated with the calcium area, and with additional characteristics indicating bifurcations and whether the analysis is performed in the main- or side-branch of the artery. The combined encodings are thereafter fed to the encoder. In the encoder, the features are first pooled and thereafter, convolutions and a transformer layer are applied. For final classification, two separate output heads are applied. In the regression head, two convolutional layers and the ReLU activation function are used. The resulting sequence is pooled along the artery dimension and subtracted from 1 to yield a single FFR value. In the classification head, the features are pooled to a fixed length of 5 (2.5 mm). Thereafter, two dense layers are used in combination with the sigmoid activation function to yield output probabilities for the presence of a functionally significant stenosis in the artery.

To assess the presence of a functionally significant stenosis, we analyze the extracted artery characteristics with a 1D convolutional neural network (Figure 5). The network performs both regression of the FFR value and classification of functionally significant stenosis. To obtain a robust final decision, we merge the predictions.

The network receives the 5 artery characteristics (lumen area, average lumen attenuation, calcium area, bifurcations and side-branches) as input. To focus on changes in lumen area and its attenuation rather than their absolute values, we calculate their percentage difference at each location in the artery with respect to the previous location. Because the relevant features in the lumen area and its attenuation may be subtle and may appear in different locations along the artery (i.e., a stenosis is expected to cause changes in the attenuation distal to the appearance in the lumen area), these two characteristics are first separately encoded. This is done using two non-shared convolutional layers with the LeakyReLU activation function applied in between the layers. Thereafter, the remaining characteristics are concatenated with the encoded features from the lumen area and its attenuation.

The information of all five extracted artery characteristics is merged by a common encoder, consisting of convolutional layers and a transformer layer, as follows: To increase the receptive field and reduce the dimensionality, average pooling with kernel size 4 is applied, followed by two convolutional layers with dilation 1 and 2, respectively. Each convolutional layer is followed by the LeakyReLU activation function, instance normalization and dropout. Subsequently, artery encodings are concatenated with the original lumen area and its attenuation, and fed to a transformer layer (42). Due to the global receptive field, the transformer layer connects all artery points with one another. This potentially enables modeling interaction between multiple lesions, and proximal and distal section of the artery. The network has two output heads that are each designed to perform a separate task: one performs regression of the FFR value and the other performs classification of the presence of a functionally significant stenosis in the artery. Inspired by the additive nature of sequential flow resistances, the regression head is designed to predict pressure drops along the artery. First, two layers of convolutions are applied, each followed by the LeakyReLU activation function, instance normalization and dropout. Thereafter, a third convolutional layer with a single output filter map is followed by a ReLU activation function to enforce positivity of the pressure drops. Finally, the predicted pressure drops are summed up along the artery using a sum pooling layer and the resulting overall FFR drop is transformed into the final FFR value by subtracting it from 1. The classification head predicts the presence of functionally significant stenosis (FFR ≤ 0.8). To explicitly relate proximal and distal sections, first, adaptive sum pooling with 5 output features is applied followed by 2 dense layers, each with LeakyReLU activation and dropout. At last, a dense layer with a single output filter map and sigmoid activation yields output probabilities for functionally significant stenosis.

For all convolutions throughout the network for stenosis assessment, a kernel size of 3 is employed in combination with zero-padding to prevent shrinkage of the features. Furthermore, for all convolutions as well as for the transformer, a relatively small number of 16 filter maps is utilized, to balance the required expressiveness and to prevent overfitting. For the same purpose, all dropout probabilities are set to 0.5.

During training, the regression head is supervised using the mean squared error with the reference FFR value. Since the invasive reference FFR is often not measured at the most distal location, predicted pressure drop contributions from anatomical locations distal to the measurement location are masked during training and testing. The measurement location is assumed to be 10 mm distal to the annotated lesion location, in line with measurement protocols from clinical practice. For the Test_NoCath_ data set, as no measurement was taken, the most distal clinically relevant location (lumen area > 2 mm^2^) was chosen as the measurement location. The classification task is supervised using the binary cross entropy loss function.

To combine strengths of the classification and the regression head, their outputs are merged into a single probability for the presence of a functionally significant stenosis in the artery. While the classification head directly predicts probabilities for the positive and negative class, the regressed FFR values are distributed around the threshold of 0.8. To allow their merging, the predicted FFR values are first transformed into pseudo-probabilities by linearly scaling a symmetric window around 0.8, using the following formula:


(1) $$\begin{array}{l} p_{pseudo}=\{\begin{array}{ll} 0.5-\frac{\left(FFR_{regress}-0.8\right)}{0.4}\; & \text{for }FFR_{regress}\in \left[0.6,1.0\right]\\ 1.0\; & \text{for }FFR_{regress}\in \left[0.0,0.6\right) \end{array} \end{array}$$

**Figure 6:**
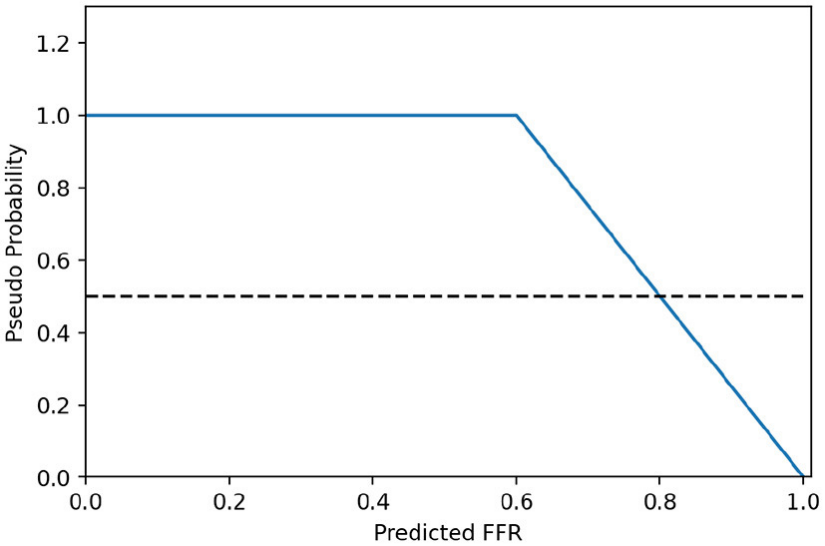
Transformation from predicted FFR values to pseudo probabilities.

Figure 6 shows the transformation function, with x values corresponding to the predicted FFR (input) and y values to the pseudo probabilities (transformations).

To obtain the final prediction result, the pseudo-probabilities are averaged with the probabilities from the classification head.

We developed our method performing randomized 10 fold cross-validation (i.e., training of 10 networks on random 90% subsets of the development selection and testing on the remaining 10%). To increase robustness of the method and determine uncertainty of the prediction, during testing we ensemble the 10 networks by averaging the predicted probabilities and FFR values (43). For the prediction of the uncertainty, we calculate the standard deviation over the probabilities and the FFR values (44).

## 4. Evaluation

We evaluate the performance of our method by computing AUC, accuracy, sensitivity, and specificity using the invasively measured FFR as reference. This is done for the final prediction, obtained by merging the classification and regression results, and for the regressed FFR values and classification probabilities separately. For evaluation, the regressed FFR values and reference FFR values were dichotomized using the threshold of 0.8 for significant stenosis. To test for the statistical significance of the AUC differences between models, we performed permutation testing (45) with 1,000 iterations and report *p*-values. To obtain a patient-level prediction, the highest output value of all classified arteries in a patient is used to assign the predicted class to the patient. In the reference, patients were considered negative if none of the measured arteries had an FFR ≤ 0.8, and otherwise positive.

## 5. Experiments and results

### 5.1. Experimental settings

To account for possible overfitting during training of the network for artery characterization, the 56 annotated arteries were split into 42 arteries for training, 4 arteries for validation and 10 arteries for quantitative testing. The network was trained utilizing the mean absolute error as loss function and the ADAMW (46) optimizer with a learning rate of 10^−5^ and a batch size of 512. Training was terminated after 800 epochs as convergence was reached. Based on preliminary experiments, the loss term of the lumen attenuation was scaled with a factor 0.1, such that all loss terms are within the same order of magnitude. After training, we applied the network to each cross-section of the MPR to obtain the lumen area, its average attenuation and the area of calcium along the length of the artery.

The network for stenosis assessment was trained for 150 epochs using the ADAMW (46) optimizer with a linearly scheduled cyclic learning rate. The cyclic learning rate varied between 5e-4 and 1e-5 over a period of 40 epochs. Because different artery lengths limit the network for stenosis assessment to process only a single artery at a time, the loss was accumulated over 8 training iterations before backpropagating, corresponding to an effective batch size of 8. The loss terms of the regression head and the classification head were weighted equally, as both terms are of similar magnitude and as both tasks are equally important.

### 5.2. Stenosis assessment

We evaluated the performance of our method on the two held out test sets, using the ensemble of 10 trained networks (Table 3). The method achieved an AUC of 0.78 on Test_Cath_ for predicting the presence of functionally significant stenosis in an artery when merging the regression and the classification. In addition, we evaluated the FFR regression and stenosis classification separately. For the Test_Cath_ data the results demonstrate that regression outperforms classification and the merged prediction, with an AUC of 0.83. On the patient level, our method achieved an AUC of 0.75 and an accuracy of 0.80.

**Table 3:** Performance of our method.

**Algorithm**	**Selection**	**AUC**	**Accuracy**	**Sensitivity**	**Specificity**
Merged	Test_Cath_	0.78	0.79	0.84	0.61
	Test_NoCath_	-	0.86	-	-
Classification	Test_Cath_	0.68	0.55	0.53	0.61
	Test_NoCath_	-	0.90	-	-
Regression	Test_Cath_	0.83	0.89	0.95	0.67
	Test_NoCath_	-	0.59	-	-

The table lists the obtained AUC, accuracy, sensitivity and specificity. Rows correspond to the classification, regression and merged outputs. To obtain binary predictions, for probabilities a threshold of 0.5 was used and for regressed FFR values a threshold of 0.8.

To investigate the performance of our method on CCTA scans without or with low degree of anatomical coronary artery stenosis, we applied the method to the Test_NoCath_ data set. Given that no scan contains functionally significant stenosis, we evaluated the performance in terms of accuracy. When merging classification and regression, the method achieved an accuracy of 0.86. The results demonstrate that for detection of arteries with little or no stenosis in the Test_NoCath_ data set, stenosis classification outperforms the FFR regression.

To assess whether the method is robust to the differences in scanner types and acquisition parameters, we investigated the performance per acquisition site on the Test_Cath_ data set (Table 4). The best performance was measured for Site 1. Note that the majority of training scans originated from this site (Table 2).

**Table 4:** Performance of our method on Test_Cath_ per site.

**Data set**	**Arteries**	**AUC**	**Accuracy**	**Sensitivity**	**Specificity**
All	76	0.78	0.79	0.84	0.61
Site 1	58	0.84	0.84	0.85	0.78
Site 2	14	0.73	0.71	0.88	0.50
Site 3	4	(0.00)	(0.25)	(0.00)	(0.33)

Results demonstrate best performance on the data from Site 1. Only 4 arteries in Test_Cath_ were acquired at Site 3 (Table 2). As this may not be sufficient to obtain a representative per-site performance, the respective numbers are presented in brackets.

**Figure 7:**
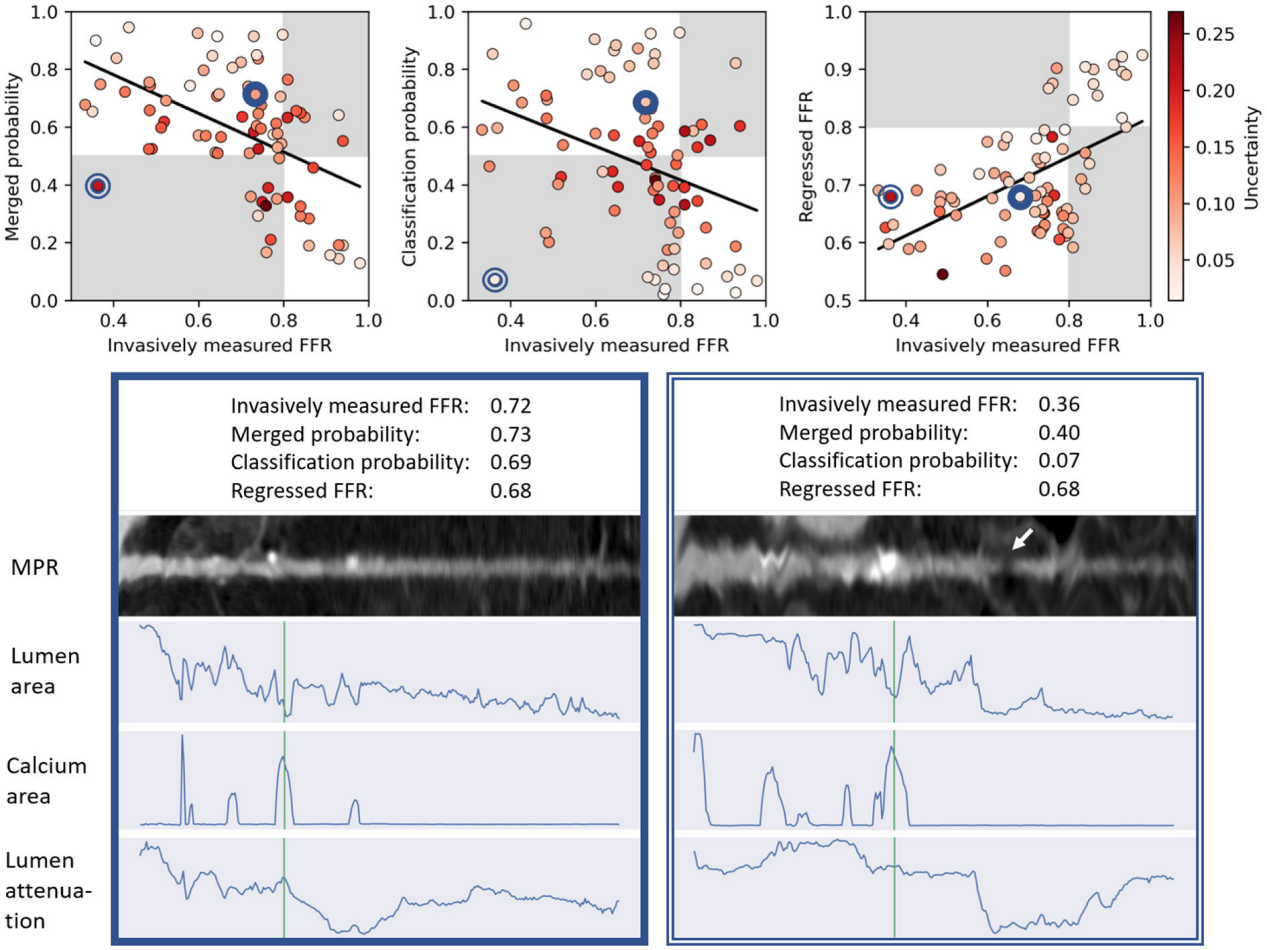
**Top:** Scatter plots relating the invasively measured FFR with the predictions for each artery from the Test_Cath_ data set. The graph on the left-hand side corresponds to the merged output probability, in the graph in the middle the output probability from the classification head is shown and in the graph on the right-hand side the regressed FFR value is depicted. Points are colored in red according to their prediction uncertainty. Background colors indicate in which arteries the functional significance was assessed correctly (white) or incorrectly (gray). Whereas, for probabilities (left and middle), high values correspond to the positive class, for regression (right), low output values correspond to the positive class. Black lines show the linear fit to the data. **Bottom:** MPRs and predicted characteristics for two arteries (positions in scatter plots indicated by blue circles). The location of the annotated lesion is plotted in green. Whereas the merged probability assigned to the artery on the left corresponds to the correct class, for the artery on the right, output of the classification head was strongly negative (low probability for functionally significant stenosis), which when combined with the regressed FFR caused the merged probability to yield the incorrect class. Incorrect output of the classification head may be related to a visually minor step-and-shot artifact causing a low intensity section in the MPR on the right (indicated by arrow).

Figure 7 shows the invasively measured reference FFR versus the merged prediction, the classification probability and the regressed FFR. The method tends to be more uncertain in incorrectly classified or regressed arteries. Furthermore, Figure 7 depicts MPRs and predicted characteristics for two arteries.

To evaluate the added value of the uncertainty measure provided by our method, we simulated a semi-automatic setting in which cases with high uncertainty are referred for invasive FFR measurement. This was done by assigning the reference FFR to the 5, 10, or 20% of cases in the Test_Cath_ data set with the highest uncertainty (Table 5). The results show that by referring 20% of the cases, a sensitivity of 0.92 with a specificity of 0.78 was reached.

**Table 5:** Performance of our method on Test_Cath_ when a percentage of cases with the highest uncertainty is excluded or corrected to the reference FFR.

		**AUC**	**Accuracy**	**Sensitivity**	**Specificity**
All arteries		0.78	0.79	0.84	0.61
Excluded	5 %	0.79	0.81	0.86	0.61
	10%	0.81	0.83	0.89	0.63
	20%	0.84	0.86	0.90	0.69
Corrected	5%	0.80	0.81	0.87	0.61
	10%	0.85	0.85	0.90	0.67
	20%	0.91	0.89	0.92	0.78

### 5.3. Contribution of artery characteristics

To determine the specific importance of each regressed characteristic, we trained and evaluated models that each only get a single characteristic as input. Additionally, the tree characteristics (bifurcation and the side-branch) were used in each network. In Table 6, the obtained performances for the Test_Cath_ data set are compared with the proposed method. The model with lumen as input performed best among the networks using only a single characteristic and the proposed method outperformed all tested models. Excluding the tree characteristics yielded a slight performance decrease.

**Table 6:** Contribution of individual characteristics.

**Characteristics**	**AUC**	**Accuracy**	**Sensitivity**	**Specificity**	***p***
All	0.78	0.79	0.84	0.61	-
No lumen	0.72	0.71	0.76	0.56	0.039
No calcium	0.73	0.54	0.52	0.61	0.152
No attenuation	0.76	0.76	0.81	0.61	0.191
No tree	0.76	0.74	0.79	0.56	0.380

Rows correspond to the proposed approach, networks trained on artery characteristic [with the characteristics from the artery tree (bifurcation and side-branch)], and a network trained on all characteristics apart from the tree characteristics (no tree). Among the separate artery characteristics, the network trained on the lumen area performed best. Including all characteristics in the proposed approach lead to the best performance. Excluding the tree characteristics resulted in a slight decrease in performance. *p*-values indicate the statistical significance of AUC improvements of the model using all characteristics over the model using the respective characteristic.

### 5.4. Comparison with previous work

Table 7 compares the performance of our method with performances of previous methods determining presence of functionally significant coronary artery stenosis, as reported in the original works. However, note that these algorithms are not publicly available, and that all of the methods were trained and tested with different proprietary data sets. Hence, the differences in the reported performance can only be seen as an indication. For each method, we indicate whether it requires the segmentation of the coronary artery at test time. The comparison shows that methods that use the artery segmentation at test time attain higher performances. However, artery segmentation is a highly challenging task and results from potentially used automatic methods require manual correction. This manual correction is a time consuming process, leading to excessive analysis times. Our method outperforms the methods that like the proposed method do not use the artery segmentation at test time.

**Table 7:** Comparison of the performance on the Test_Cath_ data set with previous work.

	**Algorithm**	**Artery segmentation**	**Analysis level**	**Analysis time**	**Samples train**	**Samples test**	**AUC**	**Accuracy**
Proposed	ML	no	Arteries	< 5 min	438	76	0.78	0.79
Proposed	ML	no	Patients	< 5 min		67	0.75	0.80
Denzinger et al. (30) ‡	ML	no	Arteries		345	*	0.88	0.87
Zreik et al. (31)	ML	no	Arteries		192	*	0.62	
Kumamaru et al. (33)	ML	no	Patients		131	*	0.69	0.71
Zreik et al. (32)	ML	no	Patients		126	*	0.74	0.70
Nørgaard et al. (8)	CFD	yes	Arteries	1–4 h	-	254	0.90	0.81
Itu et al. (21)	ML	yes	Arteries		simulations	125	0.90	0.83
Dey et al. (22)	ML	yes	Arteries		254	*	0.84	
Tesche et al. (10)	CFD	yes	Arteries	43 min	-	85	0.91	
Tesche et al. (10)	ML	yes	Arteries	41 min	Simulations	85	0.91	
Coenen et al. (11)	CFD	yes	Arteries	>30–60 min	Simulations	525	0.84	
Coenen et al. (11)	ML	yes	Arteries	>30–60 min	Simulations	525	0.84	
Ko et al. (47)	CFD	yes	Arteries		-	96	0.89	0.84
von Knebel Doeberitz et al. (23)	ML	yes	Arteries		Simulations	84	0.83	
von Knebel Doeberitz et al. (23)	ML + CFD	yes	Arteries		Simulations	84	0.93	
Wang et al. (25)	ML	yes	Arteries		Simulations	71	0.93	0.89
Gao et al. (26)	ML	yes	Arteries		Simulations	180	0.93	
Yang et al. (24)	ML	yes	Arteries		1,013	†	0.80	

*Cross-validation experiments.

†Bootstrap experiments.

‡Predicted FFR is compared to revascularization decision instead of invasive FFR.

Methods are categorized into using machine learning (ML) or computational fluid dynamics (CFD). Methods that use the artery segmentation at test time occasionally require manual interaction. Analysis times include the time needed for manual interaction.

## 6. Discussion

We presented a deep learning method that assesses the presence of functionally significant stenosis in an artery from CCTA. The method first extracts relevant characteristics from the artery's MPR by regressing the lumen area, its attenuation and the amount of calcifications, and extracting characteristics of the artery within the coronary artery tree. Subsequently, using the extracted characteristics, regression of the FFR value and classification of the presence of a functionally significant stenosis in the artery are performed and thereafter merged to obtain the final result.

The primary target population consisted of patients with an intermediate or high anatomical degree of stenosis (Test_Cath_), since these patients typically undergo invasive FFR measurement. Additionally, we investigated the performance of our method in patients with no or low degree of stenosis according to the clinically determined CAD-RADS score (Test_NoCath_). In order to make analysis in a large set feasible, we restricted evaluation to the main coronary arteries.

Results demonstrate that regression performs better in the population with intermediate or high anatomical degree of stenosis (Test_Cath_), while classification performs better in the population with low degree of anatomical stenosis or without stenosis (Test_NoCath_). To combine the strengths of both approaches and obtain robust overall performance, in this work the outputs were merged. However, in a clinical setting, the classification or regression output could be used depending on the target population. The accuracy attained on this set was higher than on Test_Cath_, demonstrating that arteries with FFR distributed around the threshold of 0.8, i.e., arteries from our primary target population, are more difficult to assess than arteries with little or no stenosis.

Literature shows that methods for predicting the presence of functionally significant stenosis from CCTA that require coronary artery segmentation achieve high performance (8, 10, 22, 24, 25, 47). However, since the performance is heavily dependent on the quality of the coronary artery segmentation, these approaches typically require manual correction of the segmentation, leading to extensive analysis times. Therefore, methods have been developed that omit the highly challenging segmentation task, leading to fast analysis. In a first investigation, Denzinger et al. (30) showed promising results for end-to-end prediction of the revascularization decision based on functional tests different from FFR in a predominantly negative population. Apart from this, methods that predict FFR without using the artery segmentation typically extract features in an unsupervised manner (31–33). These methods have not been shown to reach the same level of performance as the methods that exploit coronary artery segmentation. Hence, to incorporate information that has been shown to be important for FFR prediction (16, 20–22, 24, 26) while retaining fast analysis, we extract information directly from the MPR in a supervised manner. To do this, a limited number of artery segmentations is used to obtain reference characteristics for training a network to directly predict features characterizing the arteries at test time. During inference, our method does not require the artery segmentation and therefore, the method remains fast at inference.

While previous works used unsupervised feature extraction to describe the arteries, these features were not directly optimized to determine the FFR value (31, 32). As in previous RNN-based works (25, 26, 30), in this work extraction of features characterizing arteries and classification of the arteries are optimized together in an end-to-end fashion. However, unlike Wang et al. (25) and Gao et al. (26), we do not use pre-designed high level input features like the degree of stenosis or the lesion length. Instead, we use convolutions to locally encode the low level artery characteristics, enabling the model to learn high level features itself. Moreover, to model the interaction between proximal and distal artery segments, we include a transformer layer that enables learning global features. Furthermore, to regress the FFR value, sequential vascular resistance was modeled by adding up local pressure drops. Incorporating these inductive biases into the network enables targeted feature extraction for prediction, thereby reducing the amount of irrelevant parameters in the model. Together with a small number of descriptive characteristics per centerline point, this targeted model design mitigates the risk of potential overfitting and hence, enables end-to-end learning of high and low level features with limited training data. These features are learned using the predicted characteristics as input, which in some locations inhibit noise (see Supplementary materials). Therefore, our automatically learned features might be more robust to potential noise in the predicted characteristics than the pre-designed features used by Wang et al. (25) and Gao et al. (26). Nevertheless, training the characterization network with a larger data set of manually segmented lumen and calcium might improve the performance of our method.

To investigate the role of each characteristic, we trained additional models only on single artery characteristics. The results showed that the models using all characteristics but one reach reasonable performance and only omitting the lumen area lead to a statistically significant drop in performance (*p* < 0.05, Table 6). This is in line with previous research that underlines the importance of clinical indices derived from these characteristics for FFR prediction (14, 16). Including all characteristics in the proposed method yielded the highest performance, indicating that the extracted artery characteristics contain complementary information. Nevertheless, the proposed method is not limited to the used characteristics. Future work should investigate whether using additional characteristics, like the amount of non-calcified plaque, plaque composition, luminal diameter and artery remodeling (16, 22, 24), would further improve the performance. Furthermore, using unsupervised features (32) in addition to the targeted characteristics may be valuable as it may additionally enable extracting information that has not yet been discovered to be clinically relevant.

To identify possible causes of errors in the detection, arteries from Test_Cath_ with the largest difference between the regressed and invasively measured FFR were inspected. We found that in these arteries errors in the extraction of their characteristics were made. They frequently corresponded to overestimation of the calcium area and accordingly, underestimation of the lumen area. This indicates that although the proposed method does not model lesions explicitly, it is sensitive to errors in the artery characterizations that resemble lesions. Therefore, to further improve the performance, future work could focus on improving the artery characterizations.

By employing multiple networks in an ensemble, the robustness of our method was increased and the uncertainty of the predictions was determined (43). The uncertainty measure may be valuable in clinical practice where the method could be employed in a semi-automatic setting. In particular, patients with arteries in which the method indicates high prediction uncertainty could be referred for invasive measurements.

Separate evaluation of the method on the data from each site showed that the best performance was attained for patients scanned at Site 1. This may be caused by the fact that the training set contained most (57%) of arteries from that site. Lower performance for underrepresented sites (Site 2 and Site 3), might have been caused by differences in scanner types and acquisition parameters. Furthermore, for patients from Site 2 and Site 3, the typical time interval between the CCTA acquisition and the FFR measurement was larger compared to Site 1, which may have introduced additional noise. Another reason for performance differences between sites may relate to differences in the protocol for measuring FFR. To only account for proximal measurement positions, pressure drop contributions distal to the estimated measurement position were masked. However, the measurement location may vary between the experts and this may have caused noise in the data which may have negatively impacted performance. Using a larger, more diverse data set will likely enable improved performance for the currently underrepresented sites.

Results in this work show that when the decision threshold is optimized for high sensitivity, our method enables sparing unnecessary FFR measurement in 44% of patients with intermediate degree of stenosis while detecting 95% of functionally significant stenoses (Supplementary materials). Alternatively, combining the proposed method with expert CCTA reading may improve the performance of non-invasive detection of significant stenosis from CCTA (48). While visual assessment of CCTA by an expert radiologist has been reported to have consistently high sensitivity for detection of obstructive CAD (5, 6), it suffers from limited specificity for indicating the functional significance of a stenosis. By specifically optimizing the decision threshold, the proposed method can potentially complement the high sensitivity of expert CCTA reading with high specificity. Future work could evaluate the clinical value of automatic stenosis assessment using the proposed method in combination with expert CCTA reading.

This study has several limitations. First, a relatively small number of scans with corresponding invasive FFR measurements was retrospectively included. While data was acquired in multiple hospitals, the hospitals were not represented equally in the data set. Future work should investigate potential improvements of our method when trained on a larger dataset, equally distributed across hospitals. To avoid biases in the test data, a large-scale (prospective) study in multiple centers is required to confirm the findings. Second, 13% of patients were excluded due to lacking image quality. This may have introduced a selection bias toward patients with preferable externalities, i.e., sinus rhythm and low body-mass-index, which may have caused exclusion of patients at higher risk of significant stenosis. Third, comparison of our method with previous work can only be seen as an indication, as each method was developed and tested on different data sets. At last, we tested our trained method on arteries with no or low degree of stenosis according to the clinically determined CAD-RADS score assuming an FFR >0.8. However, it can not be fully excluded that despite the clinical stenosis assessment a small number of these arteries have FFR ≤ 0.8, e.g., due to diffuse CAD. Nevertheless, given the high sensitivity of visual assessment of CCTA for detection of CAD (5, 6), we expect this effect to be marginal.

## 7. Conclusion

We presented a deep learning approach for assessment of the functional significance of coronary artery stenosis from CCTA. Results demonstrate that the proposed approach outperforms previous works that do not require the artery segmentation as input. This indicates that the method may reduce the number of patients that unnecessarily undergo invasive measurements.

## Data availability statement

The original contributions presented in the study are included in the article/Supplementary material, further inquiries can be directed to n.hampe@amsterdamumc.nl.

## Ethics statement

The studies involving human participants were reviewed and approved by Onze Lieve Vrouwziekenhuis, Moorselbaan 164, 9300 Aalst, Belgium for Site 1; University Medical Center Utrecht, Heidelberglaan 100, 3584 CX Utrecht, Netherlands for Site 2 and University Medical Center Amsterdam, Meibergdreef 9, 1105 AZ Amsterdam, Netherlands for Site 3. Patients/participants provided their written informed consent to participate in this study (Site 1) or informed consent was waived by the respective institutional review boards (Site 2, Site 3).

## Author contributions

II and TL acquired the funding. NH, SV, and II designed the method, experiments, and drafted this manuscript. NH performed the experiments and analyzed the results. All authors critically revised this manuscript and approved the submitted version.

## Funding

This work was supported by PIE Medical Imaging BV.

## Conflict of interest

Author CC reports receiving institutional research grants from GE Healthcare, Siemens, Insight Lifetech, Coroventis Research, Medis Medical Imaging, Pie Medical Imaging, CathWorks, Boston Scientific, HeartFlow, Abbott Vascular, and consultancy fees from HeartFlow, Abbott Vascular, and Cryotherapeutics. Author II reports institutional research grants by Pie Medical Imaging, Dutch Technology Foundation with participation of Pie Medical Imaging and Philips Healthcare (DLMedIA P15-26). Author J-PA was employed by Pie Medical Imaging BV. The remaining authors declare that the research was conducted in the absence of any commercial or financial relationships that could be construed as a potential conflict of interest.

## Publisher's note

All claims expressed in this article are solely those of the authors and do not necessarily represent those of their affiliated organizations, or those of the publisher, the editors and the reviewers. Any product that may be evaluated in this article, or claim that may be made by its manufacturer, is not guaranteed or endorsed by the publisher.
