# Supplementary material for: Deep learning-based detection of functionally significant stenosis in coronary CT angiography
Source: Front Cardiovasc Med. 2022 Nov 15;9:964355. doi: 10.3389/fcvm.2022.964355 (PMC9705580; doi:10.3389/fcvm.2022.964355)
Supplement: Supplementary file 1 [file Data_Sheet_1.PDF]

Supplementary Material

1 CHARACTERIZATION NETWORK

In Figure S1 the output of the characterization network is compared to the reference for the four arteries held out for validation. Whereas for healthy segments performance of the regression of the lumen area is accurate, in the presence of pathology the prediction occasionally shows deviations from the reference. While the predicted area of calcium deviates from the reference in some lesions, the presence of calcium is predicted correctly. The average attenuation in the lumen shows a high accuracy throughout the entire artery based on visual estimation.

To additionally investigate the performance of the characterization network, we quantitatively evaluate the network using ten additional arteries randomly selected from the development dataset of the stenosis assessment network that were not used in training or validation of the characterization network. The results yielded a mean absolute error (MAE) of 0.94 mm^2 for the lumen area, 0.62 mm^2 for the calcium area and 21 HU for the average attenuation. These results are in agreement with findings from the validation set (lumen area MAE = 0.73 mm^2, calcium area MAE = 0.57 mm^2, average attenuation MAE = 16 HU) indicating that no overfitting occurred.

2 OPTIMIZING THE DECISION THRESHOLD

In clinical practice, a careful balance between sensitivity, i.e. finding all patients with functionally significant stenosis, and specificity, i.e. sparing patients without such stenosis from invasive treatment, is warranted. Whereas the threshold applied to the invasively measured FFR, i.e. 0.8, was not changed, the decision threshold used for dichotomizing the model output can be optimized to reflect this balance. Figure S2 provides insight in the performance of the method at different decision thresholds. While for $Test_{Cath}$ the accuracy decreases with rising decision thresholds, the performance increases with higher decision thresholds in $Test_{NoCath}$ due to the absence of positive samples. Table S1 provides further insight into sensitivity and specificity when varying the decision threshold. To attain a high accuracy for both selections, the threshold was set to 0.5 in all our experiments.

Table S1. Performance of our method for various decision thresholds.

Data set	Threshold	Accuracy	Sensitivity	Specificity
$Test_{Cath}$	0.30	0.84	0.95	0.44
	0.40	0.79	0.85	0.56
	0.50	0.79	0.84	0.61
	0.60	0.60	0.58	0.67
	0.70	0.54	0.42	0.94
$Test_{NoCath}$	0.30	0.53		0.53
	0.40	0.74		0.74
	0.50	0.86		0.86
	0.60	0.92		0.92
	0.70	0.96		0.96

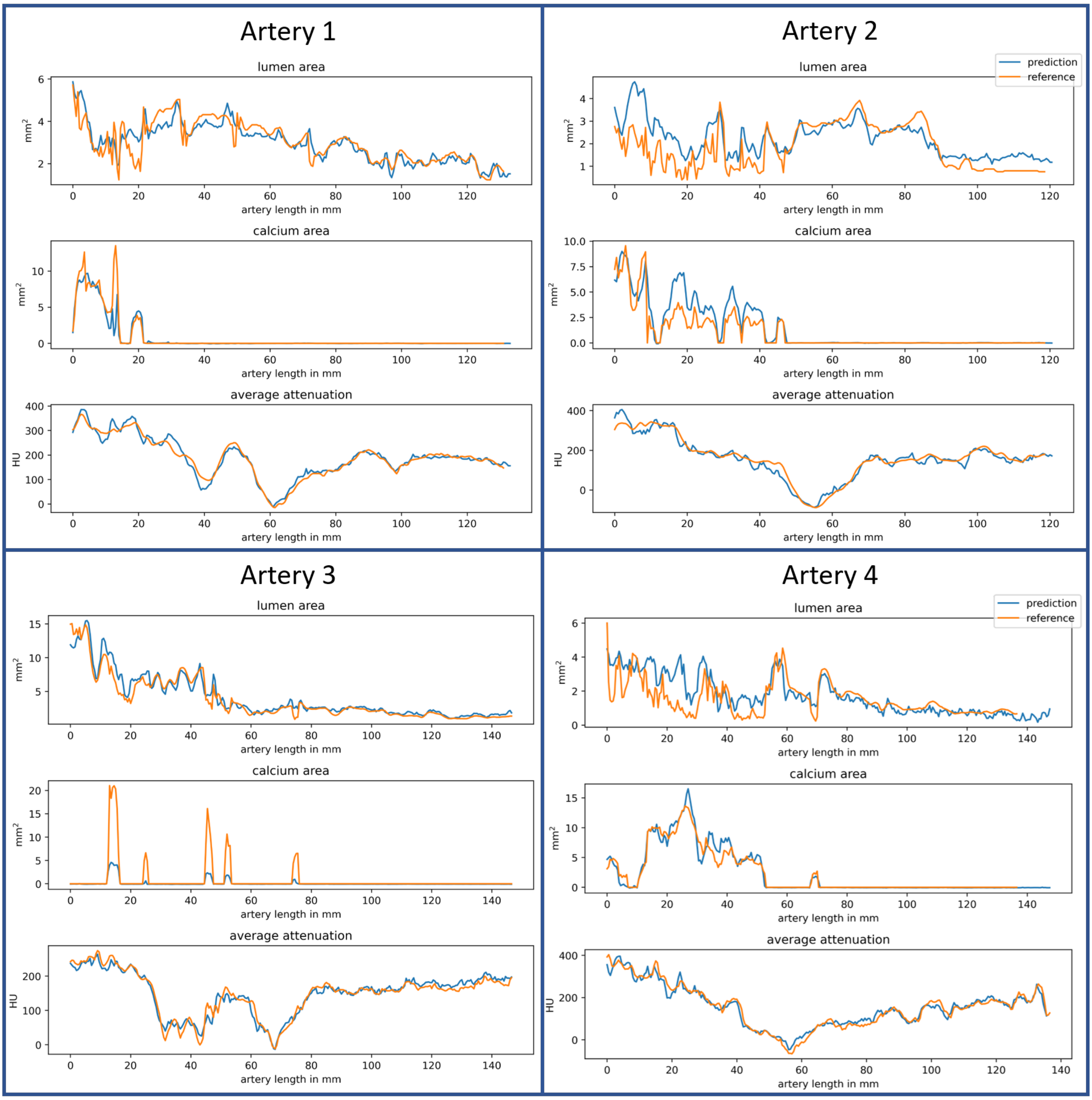

Figure S1. Comparison of predicted characteristics (blue) with the reference (orange) for the four arteries held out for validation.

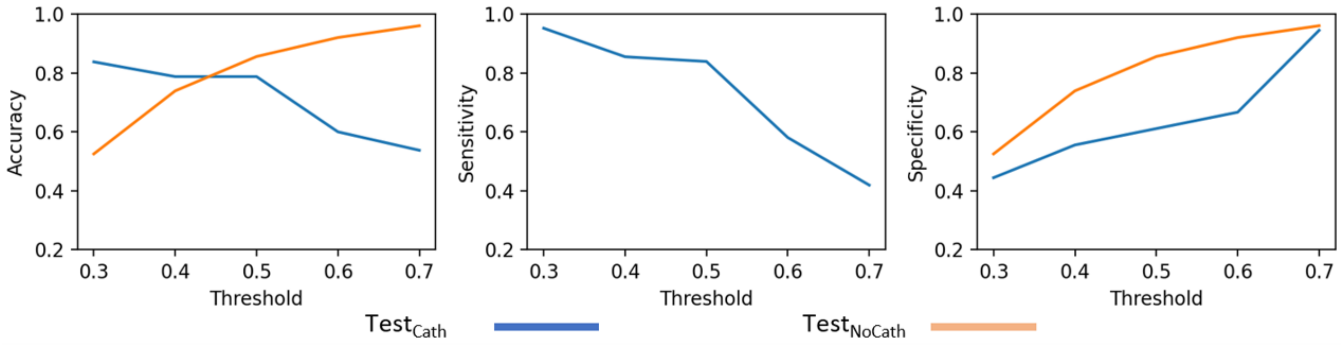

Figure S2. Optimization of the decision threshold. The figure shows accuracy, sensitivity and specificity at various decision thresholds for Test$_{\mathrm{Cath}}$ and Test$_{\mathrm{NoCath}}$. As Test$_{\mathrm{NoCath}}$ contains only negative samples, the sensitivity is undefined and the specificity equals the accuracy. Increasing the decision threshold causes an increase in the specificity and a decrease in the sensitivity. While the accuracy for Test$_{\mathrm{Cath}}$ decreases with increasing decision threshold, an increase in this threshold causes the performance on Test$_{\mathrm{NoCath}}$ to rise. To achieve a high accuracy in both selections, a decision threshold of 0.5 was chosen throughout the manuscript.
